# Obesity and carotid artery remodeling

**DOI:** 10.1038/nutd.2015.26

**Published:** 2015-08-24

**Authors:** M Kozakova, C Palombo, C Morizzo, K Højlund, M Hatunic, B Balkau, P M Nilsson, E Ferrannini

**Affiliations:** 1 Department of Clinical and Experimental Medicine, University of Pisa, Pisa, Italy; 2 Department of Surgical, Medical, Molecular Pathology and Critical Care, University of Pisa, Pisa, Italy; 3 Department of Endocrinology, Odense University Hospital, Odense, Denmark; 4 Mater Misericordiae University Hospital, Dublin, Ireland; 5 INSERM, CESP, Center for Research in Epidemiology and Population Health, U1018, University Paris Sud, Villejuif, France; 6 Department of Clinical Science, Lund University, Skåne University Hospital, Malmö, Sweden

## Abstract

**Background/Objective:**

The present study tested the hypothesis that obesity-related changes in carotid intima-media thickness (IMT) might represent not only preclinical atherosclerosis but an adaptive remodeling meant to preserve circumferential wall stress (CWS) in altered hemodynamic conditions characterized by body size-dependent increase in stroke volume (SV) and blood pressure (BP).

**Subjects/Methods:**

Common carotid artery (CCA) luminal diameter (LD), IMT and CWS were measured in three different populations in order to study: (A) cross-sectional associations between SV, BP, anthropometric parameters and CCA LD (266 healthy subjects with wide range of body weight (24–159 kg)); (B) longitudinal associations between CCA LD and 3-year IMT progression rate (ΔIMT; 571 healthy non-obese subjects without increased cardiovascular (CV) risk); (C) the impact of obesity on CCA geometry and CWS (88 obese subjects without CV complications and 88 non-obese subjects matched for gender and age).

**Results:**

CCA LD was independently associated with SV that was determined by body size. In the longitudinal study, baseline LD was an independent determinant of ΔIMT, and ΔIMT of subjects in the highest LD quartile was significantly higher (28±3 μm) as compared with those in the lower quartiles (8±3, 16±4 and 16±3 μm, *P*=0.001, *P*<0.05 and *P*=0.01, respectively). In addition, CCA CWS decreased during the observational period in the highest LD quartile (from 54.2±8.6 to 51.6±7.4 kPa, *P*<0.0001). As compared with gender- and age-matched lean individuals, obese subjects had highly increased CCA LD and BP (*P*<0.0001 for both), but only slightly higher CWS (*P*=0.05) due to a significant increase in IMT (*P*=0.005 after adjustment for confounders).

**Conclusions:**

Our findings suggest that in obese subjects, the CCA wall thickens to compensate the luminal enlargement caused by body size-induced increase in SV, and therefore, to normalize the wall stress. CCA diameter in obesity could represent an additional biomarker, depicting the impact of altered hemodynamics on arterial wall.

## Introduction

Obesity is an independent risk factor for cardiovascular (CV) disease^1, 2^ and an obesity-related increase in carotid intima-media thickness (IMT)^3, 4^ is usually interpreted as a sign of subclinical atherosclerosis. However, obesity is accompanied not only by an alteration in the metabolic profile but also by changes in systemic hemodynamics that are necessary to satisfy the metabolic demand of expanded body mass and that comprise increase in blood pressure (BP),^5, 6^ heart rate and stroke volume (SV).^7, 8, 9^ Hemodynamics has a fundamental role in controlling arterial geometry. Physiologically, arterial wall remodels in response to persistent alterations in blood flow^10, 11, 12^ and pressure,^13, 14^ thus keeping wall shear stress and circumferential wall stress (CWS) within homeostatic targets.^15^ In obesity, a body size-related increase in SV can be expected to increase luminal diameter^11, 16, 17^ in order to maintain the shear stress;^18, 19, 20^ yet an increase in luminal diameter and systemic pressure augments CWS and may induce carotid wall thickening aimed to normalize the stress.^21, 22, 23, 24^

Therefore, in the present study we tested the hypothesis that obesity-related changes in IMT might represent not only preclinical atherosclerosis but also an adaptive remodeling meant to preserve CWS in altered hemodynamic conditions. For this purpose we performed several analyses in three different populations. (A) The inter-relationships between anthropometric parameters, SV, BP, luminal diameter and IMT were studied in a healthy population with a wide range of age and body size. (B) The relationships between carotid luminal diameter, IMT and a 3-year IMT progression rate (ΔIMT) were evaluated in apparently healthy non-obese young-to-middle-aged men and women free of carotid atherosclerosis and without increased CV risk and metabolic syndrome at baseline and at 3 years. (C) Carotid IMT, luminal diameter and CWS were compared between obese subjects free of CV complications and healthy controls, matched for gender and age. The above-described relationships were studied in the proximal segment of the common carotid artery (CCA) as its simple cylindrical geometry and linear blood flow allows the application of Laplace's law for wall stress calculation.

## Materials and methods

### Study populations

Three different populations were studied: (A) Two-hundred and sixty-six apparently healthy subjects (children, adolescents and adults), free of CV disease, carotid plaques, diabetes, antihypertensive and lipid-lowering therapies, with a wide range of age (from 8 to 77 years) and body weight (from 24 to 159 kg), were recruited in a single center (Pisa).

(B) Five-hundred and seventy-one apparently healthy non-obese subjects with a low-average CV risk (assessed by the Framingham risk score) and free of the metabolic syndrome and carotid plaque at baseline and at 3 years selected from the 1566 participants of the Relationship between Insulin Sensitivity and Cardiovascular Risk (RISC) Study (www.egir.org) that recruited healthy Caucasians in 19 centers in 14 European countries. Inclusion criteria of this longitudinal study, as well as power calculation, were previously reported.^25^

(C) Eighty-eight obese subjects (children, adolescents and adults) free of CV complications and carotid plaques and 88 apparently healthy volunteers, matched for gender and age, were recruited in a single center (Pisa).

### Study protocol

The examination protocol of all three studies included medical history, anthropometry, brachial BP measurements, resting ECG, a fasting blood test, high-resolution ultrasound of extracranial carotid arteries and, for the first study population, cardiac ultrasound for SV assessment. Information regarding medical history, drug use and smoking habit was collected directly by a physician (in Pisa populations) or using standardized self-reported questionnaires (in the RISC study). A relative risk for coronary heart disease over a 10-year period was estimated from the Framingham Heart Study risk score sheets and graded as low, below average, average, above average or high.^26^ The metabolic syndrome was defined according to the US National Cholesterol Education Program Adult Treatment Panel III,^27^ as the presence of at least three out of five metabolic syndrome components (waist circumference (WC) >102 cm in men and >88 cm in women; BP ⩾130/85 mm Hg; high-density lipoprotein cholesterol <1.03 mmol l^−1^ in men and <1.29 mmol l^−1^ in women; triglycerides ⩾1.7 mmol l^−1^; fasting glucose ⩾6.1 mmol l^−1^). The study protocols conformed to the ethical guidelines of the 1975 Declaration of Helsinki Principles and was approved by local ethics committee in each center. Written consent was obtained from all participants.

### Carotid artery ultrasound imaging and analysis

Carotid ultrasound was performed according to current guidelines in all three studies.^28^ Briefly, longitudinal B-mode images of the left and right CCA, carotid bifurcation and internal carotid artery were recorded from anterior, lateral and posterior angle by a high-resolution B-mode ultrasound. The analysis of carotid images was performed using the computer-driven image analysis system MIP (Medical Image Processing; Institute of Clinical Physiology, CNR, Pisa, Italy). End-diastolic frames of the right CCA in longitudinal projection with a well-defined intima-media complex of the near and far wall were selected and digitized with a resolution of 576 × 768 pixels, and 256-degree gray scale per pixel. In the digitized image, the far-wall IMT and the luminal diameter (inner diameter, that is, distance between the lumen-intima interfaces of the near and far wall) were measured in a 1-cm long straight segment, ~1 cm before the flow divider. The value reported represents the average of three cardiac cycles. End-diastolic CWS was calculated with Lamé's equation as the product between diastolic BP (DBP in kPa) measured during image acquisition on the left brachial artery (Omron, model 705cp, Kyoto, Japan) and the ratio of end-diastolic luminal radius (*r*=diameter/2) to end-diastolic far-wall IMT (end-diastolic *r*/IMT): end-diastolic CWS (kPa)=DBP × end-diastolic *r*/IMT.^22^

In the multicenter study, carotid scans were acquired in each recruiting center by trained and certified technicians, while the analysis of carotid images was completed in a reading center (Pisa) by a single reader (MK), as previously described. Intra-observer variability of IMT and diameter measurements as well as inter-test IMT variability were tested and are previously reported.^23, 29^

### Cardiac ultrasound

SV was assessed by transthoracic Doppler-echocardiography as the product of aortic valve cross-sectional area and trans-aortic flow velocity-time integral.^9^ Trans-aortic flow was obtained in the apical projection, aortic valve opening was measured in the long-axis view and aortic valve area was calculated by circular geometry. The values used for statistical analysis are averaged over five consecutive cardiac beats. Intra-observer variability of SV measurement in our laboratory was previously reported.^23^

### Anthropometric and BP measurement

Height and weight were measured, and body mass index (BMI) was calculated as body weight (in kg) divided by squared height (in meters). In adults, obesity was defined as BMI ⩾30 kg m^−^^2^; in children and adolescents obesity was defined as BMI ⩾95th percentile of value reported for gender and age in BMI-for-age percentiles charts.^30^ WC was measured as the narrowest circumference between the lower rib margin and anterior superior iliac crest. Office brachial BP was measured by a digital electronic tensiometer (Omron, model 705cp, Kyoto, Japan, regular or large adult cuffs according to the arm circumference) in subjects seated for at least 10 min. The reported values are the mean of two measurements performed during two different visits.

### Analytical procedures

All biochemical parameters were determined by standard methods on a Roche-Modular System (Basel, Switzerland). In the multicenter study, all biochemical analyses were performed in a single center.^25^

### Statistical analysis

Data are expressed as mean±s.d., mean±s.e. and categorical data as percentages.

Variables with a skewed distribution are summarized as median and interquartile range, and log transformed for parametric statistical analyses. Analysis of covariance and Wilcoxon signed-rank test or Student's *t*-test were used to compare continuous variables, and *χ*^2^-test to compare binary variables. Relations between the outcome variables (CCA IMT, ΔIMT and luminal diameter) and continuous variables were evaluated by univariate Pearson's correlation coefficients (*r*). Multiple linear regression with backward removal (adjusted for center in the multicenter study) was used to test the independent association of outcome variables with their significant univariate correlates. Statistical tests were two-sided and significance was set at a value of *P*<0.05. Statistical analysis was performed by JMP software, version 3.1 (SAS Institute Inc., Cary, NC, USA).

## Results

### Cross-sectional study evaluating the relationship between anthropometric parameters, SV, BP, CCA luminal diameter and IMT

**Figure 1:**
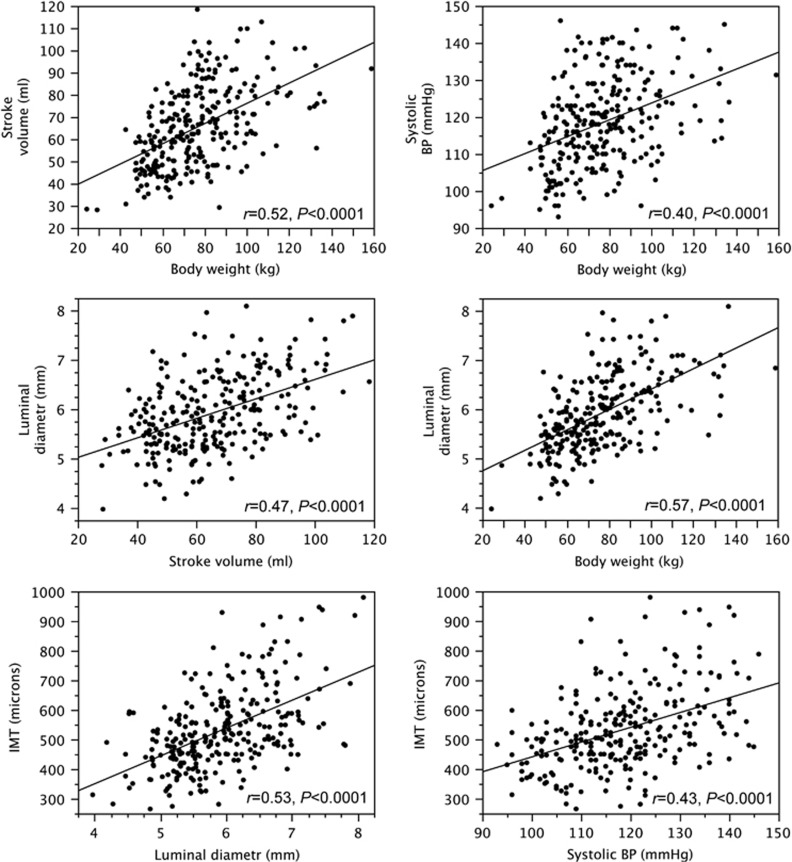
Correlations between body weight, stroke volume, blood pressure, CCA luminal diameter and IMT in 266 apparently healthy subjects.

In 266 apparently healthy subjects with a wide range of age and body weight (Supplementary Table 1), SV was directly related to body weight (Figure 1), height, BMI, WC (*r*=0.50, 0.33 and 0.28, *P*<0.0001 for all), age (*r*=0.33, *P*<0.0001), systolic BP (SBP) and DBP (*r*=0.34 and 0.20, *P*<0.0001 and *P*=0.001). SBP was directly related to body weight (Figure 1), height, BMI, WC (*r*=0.31, 0.30 and 0.30, *P*<0.0001 for all), age (*r*=0.43, *P*<0.0001) and plasma glucose (*r*=0.25, *P*<0.001). In multivariate models, following backward stepwise removal of variables, SV was determined by age, body height and body weight, and SBP was determined by male gender, age and body weight (Table 1).

CCA luminal diameter increased with SV and body weight (Figure 1), height, BMI and WC (*r*=0.43, 0.41 and 0.45, *P*<0.0001 for all), age (*r*=0.56, *P*<0.0001), SBP and DBP (*r*=0.41 and 0.38, *P*<0.0001 for both) and plasma glucose (*r*=0.26, *P*<0.0005). CCA IMT increased with age (*r*=0.73, *P*<0.0001), luminal diameter (Figure 1), SBP (Figure 1) and DBP (*r*=0.34, *P*<0.0001), low-density lipoprotein cholesterol, triglycerides and plasma glucose (*r*=0.27, 0.32 and 0.25, *P*<0.001–0.0001). In a multivariate model, luminal diameter was determined by male gender, age, WC and SV; when body weight was included in the model, it replaced SV and WC (Table 2a). CCA IMT was independently associated with age, luminal diameter and SBP (Table 2a).

### Longitudinal and cross-sectional study evaluating the relationship between baseline CCA luminal diameter and IMT or 3-year IMT progression rate

In 571 healthy non-obese subjects without increased CV risk and metabolic syndrome, BP components and established atherosclerotic risk factors remained stable during the observational period of 3 years, whereas body weight, BMI, WC and fasting plasma glucose slightly increased (Supplementary Table 2).

#### Cross-sectional data

Univariate correlates of baseline CCA IMT and luminal diameter are reported in Table 3. In a multiple regression model adjusted for centers, baseline CCA IMT was independently related to age, luminal diameter, SBP and total cholesterol, and baseline luminal diameter was independently related to male gender, age and body weight (Table 2b).

#### Longitudinal data

During a 3-year period, carotid IMT significantly increased (from 594±75 to 611±77 μm *P*<0.0001), and end-diastolic wall stress decreased (from 47.7±8.6 to 46.8±7.8 kPa, *P*<0.005). ΔIMT was directly related to age, baseline CCA diameter and plasma total and low-density lipoprotein cholesterol, and inversely to the baseline CCA IMT (Table 3), whereas it was not related to 3-year changes in atherosclerotic risk factors. In a multiple regression model adjusted for centers, the independent determinants of ΔIMT were age, baseline luminal diameter, baseline IMT and total plasma cholesterol (Table 2b).

**Figure 2:**
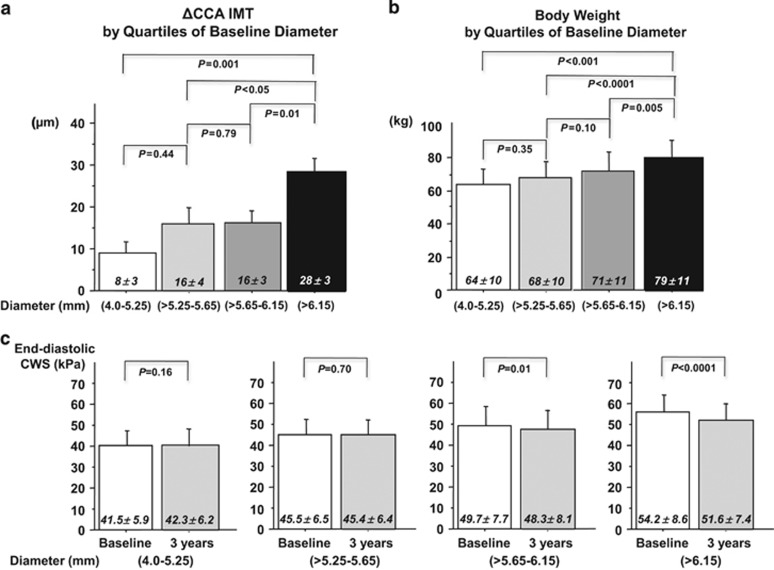
(**a** and **b**) Distribution of ΔIMT (mean±s.e.) and baseline body weight (mean±s.d.) by quartiles of baseline luminal diameter in 571 apparently healthy non-obese subjects. Statistical significance is reported after adjustment for center, sex and age. (**c**) End-diastolic circumferential wall stress (CWS; mean±s.d.) at baseline and at 3 years by quartiles of baseline luminal diameter in 571 apparently healthy non-obese subjects.

The distribution of ΔIMT, body weight and changes in CWS were evaluated also by quartiles of baseline luminal diameter. ΔIMT as well as body weight were significantly higher (*P*-value after adjustment for center, gender and age) in the highest diameter quartile as compared with lower quartiles of luminal diameter (Figure 2a and b). CWS did not change during the 3-year period in the first two quartiles of luminal diameter, whereas in the third and above all in the fourth quartile the wall stress decreased (Figure 2c).

### Case–control study evaluating CCA geometry and wall stress in obese and non-obese subjects

Carotid geometry was compared between 88 obese children, adolescents and adults without CV complications, and 88 healthy non-obese volunteers matched for gender and age (Supplementary Table 3). CCA IMT was higher in obese subjects as compared with controls, and the difference between the two groups remained highly significant (*P*=0.005) after adjustment for BP and metabolic variables (Table 4). Obese subjects had also significantly higher CCA luminal diameter and DBP (*P*<0.0001 for both), whereas end-diastolic CWS was only slightly increased (*P*=0.05).

## Discussion

Altogether, our findings suggest that in obese subjects carotid artery wall thickens to compensate the luminal enlargement and BP increase related to expansion of body mass, thus preventing a significant increase in CWS. However, our data were obtained in healthy population without increased CV risk and in obese subjects free of CV complications and may not be extrapolated to populations at higher risk or with CV disease.

Experimental studies have clearly demonstrated that arterial wall stress is maintained stable by a mutual adjustment between luminal diameter and wall thickness,^31, 32, 33^ and several clinical cross-sectional studies have shown an independent association between CCA IMT and luminal diameter.^21, 22, 23, 34, 35, 36^ We have expanded the previous findings by the observation that in a healthy non-obese population with limited impact of CV risk factors, luminal diameter was an independent determinant of IMT progression rate. In addition, subjects in the highest quartile of luminal diameter had significantly higher 3-year IMT increase as compared with those in lower quartiles, which resulted in reduction of end-diastolic CWS in this subgroup during the observational period (Figure 2).

The adjustment between arterial lumen and wall thickness is reciprocal and some studies have suggested that compensatory luminal enlargement might occur in response to arterial wall thickening.^34, 36^ Yet, in obese subjects without carotid stenosis and CV complications, the luminal enlargement that is supposed to reflect the response to body size-related increase in blood flow^11^ could be considered the initial abnormality. It has been demonstrated that a chronic increase in blood flow stimulates outward arterial remodeling aimed to maintain wall shear stress, while increasing the circumferential stress.^18, 19^ In the present study, we did not assess local carotid blood flow and wall shear stress, but we have observed a direct independent association between anthropometric parameters and SV, as well as between carotid diameter and SV or body weight, respectively (Tables 1 and 2).^9, 11^ Even in a healthy non-obese population, body weight was the strongest determinant of luminal diameter and subjects with luminal diameter in the highest quartile had significantly higher body weight (Table 2 and Figure 2).

Thus, if carotid diameter determines the changes in carotid wall thickness, the obesity-related increase in luminal diameter and BP could induce IMT thickening aimed to reduce wall stress. In our case–control study, obese subjects had highly increased both luminal diameter and BP (*P*<0.0001 for both) as compared with gender- and age-matched lean controls, yet the CWS was only slightly increased (*P*=0.05), as obese subjects had also significantly higher IMT (Table 4). These data suggest the adaptive wall thickening, and are in line with a recent study of Kappus *et al.* ^37^ showing that young obese men had increased local carotid BP, carotid diameter and IMT as compared with overweight and normal weight individuals.

The hypothesis on arterial adaptation to body size-related changes in hemodynamic load is indirectly supported by the results of several large population studies. In the Cardiovascular Risk in Young Finns Study,^38^ as well as in the Bogalusa study,^39^ the association between youth BMI and adult IMT was mediated by tracking of body mass from youth to adulthood; subjects who had been obese in youth but were non-obese as adults had IMT values comparable to subjects who had been consistently non-obese from childhood to adulthood, whereas subjects who had remained obese from childhood to adulthood had increased IMT. Furthermore, in the Cardiovascular Risk in Young Finns Study, the association between adult BMI and carotid IMT remained significant and of similar magnitude after adjustment for several metabolic risk factors, such as low-density and high-density lipoprotein cholesterol, triglycerides, insulin and C-reactive protein.^38^ In the Avon Longitudinal Study of Parents and Children,^40^ that evaluated the impact of obesity on arterial function, obese children had increased brachial artery diameter, resting and hyperemic flow and flow-mediated dilation and reduced arterial stiffness as compared with normal weight children. Observed changes were explained by physiologic adaptation to the hyperemic/hyperdynamic state of obesity.

It is worth to note the similarity between arterial and left ventricular (LV) adaptation in obesity. Obesity-related increase in circulating blood volume is associated with LV remodeling in the form of cavity dilation and compensatory LV hypertrophy, the latter representing a response to increased wall stress.^41, 42, 43, 44^ Cardiac magnetic resonance-based studies have demonstrated that over 75% of LV mass cross-sectional variation in subjects free of CV risk factors can be explained by lean mass, SV and abdominal visceral fat,^43^ and that increase in LV end-diastolic wall stress leads to LV hypertrophic response aimed to compensate for LV dilation.^44^ On the other hand, some growth factors related to obesity, such as insulin-like growth factor-1, insulin-like growth factor-binding protein-3 and fibroblast growth factor,^45, 46^ may also participate on IMT thickening and LV hypertrophy in obese subjects, as their relationship to preclinical atherosclerosis or LV mass has been recognized.^47, 48, 49^

### Study limitations

DBP used for the calculation of CWS in carotid artery was measured at brachial and not carotid artery level. However, it is known that DBP, in contrast to SBP, does not change substantially throughout the arterial tree.^50^ Wall shear stress, that could better explain the association between obesity, SV and luminal diameter, was not assessed. Only established risk factors were evaluated in our populations, and the impact of other risk factors, such as lipoproteins, adipocytokines, insulin resistance or chronic inflammation, on carotid wall thickness cannot be excluded.

## Conclusions

Results of our cross-sectional, longitudinal and case–control studies support the postulate that an increase in carotid wall thickness in obesity should not be simply interpreted as accelerated atherosclerosis, but an adaptive remodeling aimed to normalize increased CWS caused by body size-related increase in BP, SV and luminal diameter, should also be considered. Therefore, in obesity, carotid luminal diameter enlargement might represent an additional marker of CV risk that reflects the adverse impact of hemodynamic overload on vascular system. Our data also indicate that the weight reduction should be the most effective measure for reversing carotid wall thickening in obesity, as it is the only way to normalize central resting hemodynamics.^9, 51^

## Figures and Tables

**Table 1:** Independent determinants of stroke volume and systolic blood pressure in apparently healthy population with wide range of age and body weight

		*β*±s.e.*	P-*value*
Stroke volume (ml)	Age (years)	0.17±0.05	0.001
	Body weight (kg)	0.36±0.05	<0.0001
	Body height (m)	0.28±0.06	<0.0001
Cumulative *R*^2^		0.38	<0.0001
Systolic BP (mmHg)	Gender (male)	0.15±0.05	<0.01
	Age (years)	0.36±0.05	<0.0001
	Body weight (kg)	0.31±0.05	<0.0001
Cumulative *R*^2^		0.31	<0.0001

*β=standardized regression coefficient.

**Table 2:** Independent determinants of CCA IMT, ΔIMT and luminal diameter

	*Baseline CCA IMT (μm)*		*ΔCCA IMT (μm)*		*Baseline luminal diameter (mm)*	
	*β*±s.e.*	P-*value*	*β*±s.e.*	P-*value*	*β*±s.e.*	P-*value*
a) *Healthy population with wide range of age and body size;* N*=266*						
Gender (male)					0.14±0.05	0.005
Age (years)	0.58±0.05	<0.0001			0.42±0.05	<0.0001
Waist circumference (cm)					0.25±0.05	<0.0001
Stroke volume (ml)					0.19±0.05	<0.0001
Baseline luminal diameter (mm)	0.15±0.05	<0.01				
Systolic BP (mm Hg)	0.12±0.05	0.01				
Cumulative *R*^2^	0.56	<0.0001			0.51	<0.0001
Gender (male)					0.16±0.04	<0.0001
Age (years)					0.45±0.04	<0.0001
Body weight (kg)					0.43±0.04	<0.0001
Cumulative *R*^2^					0.57	<0.0001
						
b) *Healthy non-obese population without increased cardiovascular risk;* N*=571*						
Gender (male)					0.26±0.04	<0.0001
Age (years)	0.44±0.04	<0.0001	0.26±0.04	<0.0001	0.12±0.04	0.001
Baseline luminal diameter (mm)	0.19±0.04	<0.0001	0.24±0.04	<0.0001		
Baseline CCA IMT (μm)			−0.43±0.04	<0.0001		
Body weight (kg)					0.29±0.04	<0.0001
Systolic BP (mm Hg)	0.09±0.04	0.01				
Total cholesterol (mmol l^−1^)	0.11±0.04	<0.005	0.12±0.04	<0.005		
Cumulative *R*^2^	0.36	<0.0001	0.25	<0.0001	0.32	<0.0001

Abbreviations: BP, blood pressure; CCA, common carotid artery; IMT, intima-media thickness.

*β=standardized regression coefficient.

**Table 3:** Univariate correlations between carotid structural measures, anthropometric parameters and risk factors in 571 non-obese healthy subjects

	*Baseline CCA IMT (μm)*	*ΔCCA IMT (μm)*	*Baseline luminal diameter (mm)*
Baseline CCA IMT (μm)	—	−0.22	0.23
Baseline diameter (mm)	0.23	0.17	—
Age (years)	0.47	0.14	0.12
BMI (kg m^−^^2^)	0.18	NS	0.30
Body weight (kg)	0.15	NS	0.47
Waist circumference (cm)	0.22	NS	0.42
Systolic BP (mm Hg)	0.21	NS	0.23
Diastolic BP (mm Hg)	0.16	NS	0.13
Total cholesterol (mmol l^−1^)	0.27	0.13	NS
LDL-cholesterol (mmol l^−1^)	0.27	0.10	NS
HDL-cholesterol (mmol l^−1^)	NS	NS	−0.19
Triglycerides (mmol l^−1^)	0.15	NS	0.15
Fasting glucose (mmol l^−1^)	0.13	NS	0.29
Fasting insulin (pmol l^−1^)	NS	NS	NS

Abbreviations: BP, blood pressure; BMI, body mass index; CCA, common carotid artery; HDL, high-density lipoprotein; IMT, intima-media thickness; LDL, low-density lipoprotein; NS, not significant.

**Table 4:** CCA IMT, luminal diameter, circumferential wall stress in non-obese and in obese subjects matched for gender and age

	*Non-obese*	*Obese*	P*-value*	P*-value* *after adjustment*
CCA IMT (μm)	516±89	615±122	<0.0001	0.0005 adjustment for BP
				0.001 adjustment for BP and plasma lipids
				0.005 adjustment for BP, plasma lipids and FPG
Luminal diameter (mm)	5.53±0.48	6.23±0.71	<0.0001	
Diastolic BP (mm Hg)	70±8	78±11	<0.0001	
End-diastolic CWS (kPa)	50.8±9.4	53.8±13.3	0.05	

Abbreviations: BP, blood pressure; CCA, common carotid artery; CWS, circumferential wall stress; FPG, fasting plasma glucose

; IMT, intima-media thickness.
