# Supplementary material for: Obesity and carotid artery remodeling
Source: Nutr Diabetes. 2015 Aug 24;5(8):e177–. doi: 10.1038/nutd.2015.26 (PMC4558557; doi:10.1038/nutd.2015.26)
Supplement: Supplementary Table 1 [file nutd201526x1.docx]

**Table 1 Supplemental** **Characteristics of 266 Apparently Healthy Subjects with Stroke Volume Measurement**

|  | ***mean±SD/***  ***median[IQR]*** | **Range** |
| --- | --- | --- |
| Male:Female | 132:134 |  |
| Age (years) | 33±19 | 8-77 |
| Weight (kg) | 76±20 | 24-159 |
| Height (m) | 166±11 | 127-191 |
| BMI (kg/m^2^) | 27.6±6.6 | 15.1±50.5 |
| Waist circumference (cm) | 94±15 | 46-150 |
| Systolic BP (mmHg) | 118±12 | 93-146 |
| Diastolic BP (mmHg) | 71±9 | 50-92 |
| HDL-cholesterol (mmol/L) | 1.3±0.3 | 0.8-2.5 |
| LDL-cholesterol (mmol/L) | 2.8±0.7 | 1.0-4.1 |
| Triglycerides (mmol/L) | 0.9[0.7] | 0.5-2.3 |
| Fasting glucose (mmol/L) | 5.0±0.7 | 3.4-6.3 |
| Current smoking (%) | 11.7% |  |
|  |  |  |
| Stroke volume (ml) | 65±18 | 28-118 |
| CCA luminal diameter (mm) | 5.92±0.76 | 4.0-8.1 |
| CCA IMT (μm) | 530±135 | 270-980 |
