# Supplementary material for: Obesity and carotid artery remodeling
Source: Nutr Diabetes. 2015 Aug 24;5(8):e177–. doi: 10.1038/nutd.2015.26 (PMC4558557; doi:10.1038/nutd.2015.26)
Supplement: Supplementary Table 2 [file nutd201526x2.docx]

# Table 2 Supplemental Characteristics of the Longitudinal Study Population

|  | **Baseline** | **3-year** | ***P**** |
| --- | --- | --- | --- |
|  | ***mean±SD/***  ***median[IQR]*** | ***mean±SD/***  ***median[IQR]*** |  |
| Male/Female | 241:330 |  |  |
| Age (years) | 44±8 |  |  |
| Weight (kg) | 70.8±11.6 | 71.0±11.9 | <0.005 |
| Waist circumference (cm) | 83±10 | 84±11 | <0.01 |
| BMI (kg/m^2^) | 24.1±2.7 | 24.3±2.9 | 0.001 |
| Systolic BP (mmHg) | 115.8±11.6 | 116.4±11.9 | 0.11 |
| Diastolic BP (mmHg) | 73.2±7.5 | 73.4±7.8 | 0.59 |
| Heart rate (bpm) | 67±10 | 68±9 | <0.05 |
| Total cholesterol (mmol/L) | 4.85±0.82 | 4.88±0.85 | 0.22 |
| LDL-cholesterol (mmo/L) | 2.88±0.76 | 2.88±0.78 | 0.74 |
| HDL-cholesterol (mmo/L) | 1.51±0.39 | 1.52±0.43 | 0.15 |
| Triglycerides (mmol/L) | 0.88[0.53] | 0.88[0.51] | 0.73 |
| Fasting glucose (mmo/L) | 5.05±0.51 | 5.18±0.56 | <0.0001 |
| Fasting insulin (pmo/L) | 27[20] | 27[17] | 0.10 |
| Current smoking (%) | 24.9 | 19.7 | <0.0001 |

*: statistical significance between values at baseline and at 3 years.
