# Supplementary material for: Obesity and carotid artery remodeling
Source: Nutr Diabetes. 2015 Aug 24;5(8):e177–. doi: 10.1038/nutd.2015.26 (PMC4558557; doi:10.1038/nutd.2015.26)
Supplement: Supplementary Table 3 [file nutd201526x3.docx]

**Table 3 Supplemental** **Characteristics of 88 Non-obese Healthy Subjects and 88 Obese Subjects Free of Cardiovascular Disease Matched for Gender and Age**

|  | **Non-obese** | **Obese** | ***P*** |
| --- | --- | --- | --- |
| Male:Female | 45:43 | 45:43 |  |
| Age (years) | 38±16 | 38±16 |  |
| Weight (kg) | 65±11 | 107±19 | <0.0001 |
| BMI (kg/m^2^) | 22.9±2.5 | 37.3±6.0 | <0.0001 |
| Waist circumference (cm) | 82±10 | 115±17 | <0.0001 |
| Systolic BP (mmHg) | 113±11 | 126±14 | <0.0001 |
| Diastolic BP (mmHg) | 70±8 | 78±11 | <0.0001 |
| Heart rate (bpm) | 64±10 | 73±12 | <0.0001 |
| HDL-cholesterol (mmol/L) | 1.5±0.4 | 1.2±0.3 | <0.0001 |
| LDL-cholesterol (mmol/L) | 2.7±0.7 | 3.0±0.7 | <0.01 |
| Triglycerides (mmol/L) | 0.8[0.5] | 1.3[1.0] | <0.0001 |
| Fasting glucose (mmol/L) | 4.9±0.6 | 5.7±1.5 | <0.0005 |
| Current smoking (%) | 13.6 | 14.8 | 0.83 |
| Diabetes mellitus (%) | 0 | 13.6 |  |
| High blood pressure (%) | 0 | 21.5 |  |
